# Efficiency and safety of vitrification of surplus oocytes following superovulation: a comparison of different clinical indications of oocyte cryopreservation in IVF/ICSI cycles

**DOI:** 10.3389/fendo.2023.1221308

**Published:** 2023-10-05

**Authors:** Xiao Fu, Yiting Zhang, Shuli Gao, Shuzhe Gao, Meng Zhang, Shanshan Gao, Jinlong Ma, Zi-Jiang Chen

**Affiliations:** ^1^Center for Reproductive Medicine, the Second Hospital, Cheeloo College of Medicine, Shandong University, Jinan, Shandong, China; ^2^Key laboratory of Reproductive Endocrinology of Ministry of Education, Shandong University, Jinan, Shandong, China; ^3^Shandong Key Laboratory of Reproductive Medicine, Jinan, Shandong, China; ^4^Shandong Provincial Clinical Research Center for Reproductive Health, Jinan, Shandong, China; ^5^Shandong Technology Innovation Center for Reproductive Health, Jinan, Shandong, China; ^6^National Research Center for Assisted Reproductive Technology and Reproductive Genetics, Shandong University, Jinan, Shandong, China; ^7^Shanghai Key Laboratory for Assisted Reproduction and Reproductive Genetics, Shanghai, China; ^8^Center for Reproductive Medicine, Ren Ji Hospital, School of Medicine, Shanghai Jiao Tong University, Shanghai, China

**Keywords:** autologous oocyte vitrification, survival rate, live birth, sperm, cumulative live birth rate

## Abstract

**Objective:**

To evaluate the effectiveness and safety of utilizing the small number of remaining vitrified oocytes after the failure of adequate fresh sibling oocytes. The outcome of present study would provide more comprehensive information about possible benefits or disadvantage to cryopreserve supernumerary oocytes for patients who have plenty oocytes retrieved.

**Methods:**

This retrospective cohort study included 791 IVF/ICSI cycles using 6344 oocytes that had been vitrified in the Reproductive Hospital affiliated to Shandong University between January 2013 and December 2019.They were divided into three groups: SOC group (supernumerary oocytes cryopreservation), relative-MOC group (relative male factor-oocyte cryopreservation), and absolute-MOC group (absolute male factor-oocyte cryopreservation). Laboratory and clinical outcomes were analysed, and multivariate regression analysis was used to study the effect of different indications of vitrification on CLBR.

**Results:**

The CLBR was highest in absolute-MOC, and lowest in SOC (39.0% vs 28.9%, P=0.006); however, after adjusting for confounding factors, the difference was not statistically significant. Multivariable regression analysis showed no impact of indications of vitrified oocytes on CLBR according to controlled age, BMI, preservation duration, use of donor sperm or not, use of PESA/TESA or not, number of oocytes retrieved, number of oocytes thawed, and oocyte survival rate. The preliminary data of safety showed no significant differences in the perinatal and neonatal outcoms after ET and FET between the SOC and MOC groups.

**Conclusion:**

Different indications of vitrification did not affect CLBR. The CLBR of vitrified oocytes for different indications was correlated with age and number of warmed oocytes. For women who have plenty oocytes retrieved, the strategy of cryopreserving a small number of oocytes is a valuable option and might benefit them in the future. Additional data from autologous oocyte vitrification research employing a large-scale and variable-controlled methodology with extending follow-up will complement and clarify the current results.

## 1 Introduction

Vitrification is now an established method of oocyte preservation and has replaced the traditional method of slow freezing (1). Nowadays, oocyte vitrification is a viable strategy for different clinical indications, such as unexpected unavailability of sperm at the time of oocyte retrieval (2), reducing the portion of the embryos initially created, elective fertility preservation (FP), and FP before cancer treatment (3). Another indication for oocyte vitrification is the establishment of donor oocyte banks (1, 4, 5). In mainland China, supernumerary oocytes are commonly vitrified for future use in IVF/ICSI cycles when more than the required number of oocytes are retrieved. On the one hand, it provides different strategies for FP (embryo and oocytes preservation) to women with supernumerary oocytes, and on the other hand, this facilitates the creation of donor oocyte banks through an oocyte-sharing program (4).

The recommendation to cryopreserve supernumerary oocytes for women with more than 18–20 oocytes, is based on a large body of evidence. Neves et al. showed that the available evidence suggests that the number of oocytes is strongly associated with the CLBR (6). Sunkara et al. showed that the highest pregnancy rate for a single cycle was obtained with 15 oocytes (7). Fanton and his colleagues found the CLBR increased rapidly with the number of oocytes retrieved to approximately 16-20 oocytes, at which point it continued to increase but with diminishing returns (8). Therefore, in a cycle with excessive oocyte retrieval (>20), vitrification of a small number of oocytes (≥3) and is recommended, and this strategy might hardly decreased the chance of live birth for a single cycle with the remaining at least 15 fresh oocyte for fertilization. These women generally retain their vitrified oocytes until they have had a live birth. Then, in our centre, most patients would continue to store cryopreserved oocytes for their own use, some would donate these oocytes, and some would discard them. Between 2013 and 2019, 4536 IVF/ICSI cycles involving the freezing of supernumerary oocytes were performed at our reproductive centre. During the same period, only 691 women donated their vitrified oocytes for other patients.

However, some women returned to use their autologous vitrified oocytes, mostly because they had not achieved a live birth from the more than 15 fresh oocytes, owing to inferior embryos or implantation failures. A concern for both the patients and clinicians is the value of using autologous cryopreserved oocytes in cases of the failure of fresh sibling oocytes from the same retrieval cycles, and whether these oocytes are inherently less viable or less safe than oocytes frozen for other indications.

Therefore, we compared these supernumerary vitrified-warmed oocytes (SOC, supernumerary oocytes cryopreservation) cycles with oocyte warming cycles for male reasons (MOC, male factor-oocyte cryopreservation). The aim of this study was to evaluate the effectiveness and safety of utilizing the small number of remaining vitrified oocytes after the failure of adequate fresh sibling oocytes. The outcome of the present study offers comprehensive information regarding the possible benefits or disadvantages of cryopreserving supernumerary oocytes for patients with a surplus of oocytes after retrieval.

## 2 Materials and methods

This is a retrospective cohort study conducted at the Reproductive Hospital of Shandong University. The ethics committee at the Reproductive Hospital of Shandong University approved the study protocol.

### 2.1 Study design and population selection

Data of all autologous IVF/ICSI cycles using oocytes that had been vitrified in our reproductive centre between January 2013 and December 2019 were extracted. These oocytes had previously been vitrified between 2008 and 2019. Women in this cohort were undergoing medically recommended IVF/ICSI with oocyte cryopreservation owing to the collection of an excessive number of oocytes (>18–20), or because of lack of sperm on oocyte retrieval day (male partner had none or insufficient sperm, an inability to provide an ejaculated sample, or their unexpected absence on oocyte retrieval day). According to the indications of oocyte vitrification, the cycles were divided into three group: SOC group (infertile couples with an excess number of oocytes), relative MOC group (owing to the male partner being unable to provide an ejaculated sample through masturbation or their unexpected absence on retrieval day), and absolute MOC group (owing to the male partner being unable to produce, or there being insufficient sperm from an ejaculated sample or surgical collection on oocyte retrieval day).

### 2.2 Oocyte vitrification/thawing method

After incubation at 37°C and 6% CO_2_ for 3–4 h, oocytes intended for vitrification were placed in hyaluronidase medium (SAGE BioPharma, NJ, USA) to remove coronal cells. Only mature oocytes were vitrified. Three vitrification kits were used in this study, including two commercially available kits, the so-called MC kit and the KT kit, and a Modified kit prepared in our laboratory. The penetrating cryoprotectants in the MC kit were ethylene glycol (EG) and 1,2-propanediol (PROH). The KT kit included EG and dimethyl sulphoxide (DMSO). The Modified kit was made up of three penetrating cryoprotectants: EG (Sigma-Aldrich, St. Louis, MO, 102466, USA), DMSO (Sigma-Aldrich, St. Louis, MO, D2650, USA), and PROH (Sigma-Aldrich, St. Louis, MO, 544324-068, USA). Materials used to prepare the vitrification kit include equilibrium solution (ES) and vitrification solution (VS). As for the MC kit and KT kit, ES included 7.5% EG + 7.5% PROH (DMSO), and VS included 15% EG + 15% PROH (DMSO) + 0.5 mol/L sucrose, per the instructions. The Modified kit was prepared with M-199 (Gibco Invitrogen Corp., Grand Island, NY, USA) as the basal media. A 20% serum plasma substitute (SPS) (SAGE, Trumbull, CT, USA) was also added. For the Modified kit, ES comprised 7.5% EG + 3.75% DMSO + 3.75% PROH, and VS comprised 15% EG + 7.5% DMSO + 7.5% PROH + 0.5 mol/L sucrose in a M-199 medium with 20% SPS (9).

Oocytes intended for vitrification were equilibrated at room temperature (RT) in ES for 5–10 min until they recovered their shape, and then they were moved to the VS for 1 min. Finally, the oocytes were placed on a CryoLoop (Hampton Research, Laguna Niguel, CA, USA) and immediately immersed in liquid nitrogen. No more than four oocytes were loaded onto each CryoLoop. Oocyte warming was performed at RT, except for the first step. The CryoLoop with the vitrified oocytes was taken out of the liquid nitrogen and immediately placed in 1.0 mol/L sucrose in a M-199 + 20% SPS solution at 37 °C for 1.5–2.0 min. Oocytes were then placed in 0.5 mol/L sucrose in an M-199 + 20% SPS solution for 3 min at RT, after which they were transferred into another M-199 solution with 0.25 mol/L sucrose for 3 min. Finally, they were washed in M-199 + 20% SPS for 5–10 min, while the stage was warmed slowly. After warming, the surviving oocytes were cultured for 2 h in G-IVF (Vitrolife, Göteborg, Sweden) in an incubator with 37 °C, 6% CO_2_ before being inseminated via ICSI  (9).

### 2.3 Intracytoplasmic sperm injection and embryo culture

All viable oocytes underwent ICSI for fertilization. Fertilization was evaluated 16–18 h later, and embryos with two pronuclei were regarded as normal fertilized embryos on Day 1. Embryos with 3–5 uniformly sized blastomeres on day 2 (40–42 h after injection) and more than 7 uniformly sized blastomeres on day 3 (64–66 h after injection) were regarded as normal developing embryos. According to Puissant’s standard for embryo grades, >7 blastomeres and <20% fragments on Day 3 are deemed high-quality embryos. According to Gardner’s standard (10), only blastocysts evaluated with grade 4BC and higher quality should be cryopreserved. The number and stage of embryo transfer depends on the age, parity, medical history, embryo quality, and the patients’ decision. No more than 2 embryos were included per transfer.

### 2.4 Endometrial preparation and pregnancy assessment

All patients used hormone replacement therapy. The endometrial preparation protocols in our reproductive medicine centre have been described in detail elsewhere (11). Briefly, the women took 4–8 mg of oral estradiol valerate (Progynova, Bayer, Germany) daily for at least 10 d starting on day 2–5 of the menstrual cycle. When the endometrial thickness reached ≥8 mm, oral progesterone (Dydrogesterone, Solvay, the Netherlands) 20 mg twice daily plus vaginal micronized progesterone (Utrogestan, Besins Manufacturing Belgium) 200 mg once daily were initiated on the day of oocyte warming. Clinical pregnancy was determined as the presence of an intrauterine gestational sac identified with positive cardiac movement on ultrasound 4–5 weeks after embryo transfer (ET). Live birth was defined as the delivery of a live-born infant after 28 weeks of gestation. Early miscarriage was defined as the spontaneous loss of a clinical pregnancy within the first 13 weeks of gestation.

### 2.5 Statistical analysis

The primary outcome was the cumulative live birth rate (CLBR) defined as the delivery of a live born (>24 weeks of gestation) per warming cycle, including live birth from fresh ETs and subsequent cryo-ETs. The secondary outcome included survival rate, laboratory outcomes of vitrified-warmed oocytes, clinical pregnancy rates, first trimester abortion rate, and the live birth rate per fresh embryo transfer and vitrified embryo transfer, perinatal outcomes, as well as neonatal outcomes.

Continuous variables were presented as mean ± standard deviation. The differences between groups were analysed by independent sample t-test if data were normally distributed, otherwise it was analysed using the Kruskal-Wallis test. Categorical variables were presented as frequencies and percentages, and they were analysed by chi-square test. Multivariate logistic regression analysis was used to identify characteristics that may be associated with the CLBR. The age, body mass index (BMI), indications for oocyte freezing, preservation duration, donor frozen sperm, husband PESA/TESA sperm, number of oocytes retrieved, number of thawed oocytes, and vitrified oocyte survival rate were included in the analysis. The oocyte-to-baby rate was calculated by dividing the number of live births by the total number of oocytes consumed×100. The cumulative probability of live birth (CLBR) was estimated by the Kaplan-Meier method based on the total number of oocytes thawed in consecutive procedures, including oocytes from cancelled ETs and from fresh or cryo-ETs, until a live birth was achieved. All statistical analyses were performed with the Statistical Package for the Social Sciences (v.26.0; SPSS Inc., Chicago, IL, USA). A P-value of <0.05 was considered statistically significant.

## 3 Results

A total of 6344 vitrified oocytes warmed for 791 cycles were included in the study; they were divided into a SOC group (n=429), a relative-MOC group (n=90), and an absolute-MOC group (n=272) dependent upon indications for oocyte vitrification.

The baseline characteristics of the three groups with different indications for oocyte vitrification and the pregnancy outcomes of fresh sibling oocytes in the same oocytes retrieved cycle are shown in **Table 1**. There were significant differences between the three groups in many variables, including age, BMI, follicle-stimulating hormone (FSH), luteinizing hormone (LH), testosterone, ovulation stimulation protocols, the number of oocytes retrieved, the number of vitrified oocytes, and preservation duration. In relative-MOC, all oocytes retrieved were vitrified; and in SOC and absolute-MOC, oocytes were part-vitrified or all-vitrified. No significant differences in clinical pregnancy rate, early miscarriage rate, or live birth rate were observed between SOC and absolute-MOC in fresh embryo transfers from fresh oocytes. The clinical pregnancy rate was remarkably higher in SOC than that in absolute-MOC (41.1% vs 11.1%, P=0.012) in frozen embryo transfers from fresh oocytes. However, only 42 and 4 live births were born from fresh oocytes in SOC and absolute-MOC, and the live birth rates were 9.80% and 6.35% per cycle, respectively.

**Table 1:** Baseline characteristics of patients with different indications for oocyte freezing.

	SOC (*n* = 429)	Relative MOC (*n* = 90)	Absolute MOC(*n* = 272)	*p* value^①^	*p* value^②^	*p* value^③^
Age, (y)^ac^	29.56±3.84	35.10±5.84	30.20±4.97	**<0.001**	0.072	**<0.001**
BMI, (kg/m2) ^bc^	23.86±3.72	24.44±3.57	23.10±3.63	0.176	**0.008**	**0.002**
Basal Hormones						
FSH (IU/L) ^abc^	5.88±1.42	7.21±2.47	6.47±2.01	**<0.001**	**<0.001**	**0.011**
LH (IU/L) ^ab^	6.45±4.55	5.19±2.76	5.38±2.81	**0.001**	**<0.001**	0.573
To (ng/dl) ^abc^	31.69±17.26	21.42±13.15	26.62±13.17	**<0.001**	**<0.001**	**0.001**
Ovulation stimulation protocols,n(%) ^abc^				**<0.001**	**<0.001**	**<0.001**
Ultra long protocol	18(4.2)	4(4.4)	8(2.9)			
Long protocol	255(59.4)	26(28.9)	162(59.6)			
Short protocol	15(3.5)	34(37.8)	53(19.5)			
Antagonist	92(21.4)	18(20)	46(16.9)			
Others	49(11.4)	8(8.9)	3(1.1)			
Oocytes retrieved ^abc^	22.02±7.86	8.66±5.48	13.43±6.26	**<0.001**	**<0.001**	**<0.001**
Frozen oocytes ^bc^	7.61±3.83	7.69±4.75	11.32±6.27	0.880	**<0.001**	**<0.001**
Preservation duration(m) ^abc^	6.97(3.60-12.85)	2.85(2.00-4.00)	4.10(2.83-7.15)	**<0.001**	**<0.001**	**0.002**
Fresh oocyte cycle, n(%) ^abc^				**<0.001**	**<0.001**	**<0.001**
All-oocyte-vitrified cycles	0	90(100)	209(76.8)			
Part-oocyte-vitrified cycles	429(100)	0	63(23.2)			
Fresh embryo transfer cycles	n=133		n=24			
Clinical pregnancy rate, n(%)	19(14.3)	–	2(8.3)	–	0.644	–
Early miscarriage rate, n(%)	9(6.8)	–	0	–	0.403	–
Live birth rate, n(%)	5(3.8)	–	2(8.3)	–	0.644	–
Frozen embryo transfer cycle	n=241		n=18			
Clinical pregnancy rate, n(%)^ac^	99(41.1)	–	2(11.1)	–	**0.012**	–
Early miscarriage rate, n(%)	55(55.6)	–	0	–	0.205	–
Live birth rate, n(%)	37(15.4)	–	2(11.1)	–	0.886	–
Abandoned transplant cycles*,n ^ac^	88	–	24	–	**<0.001**	–

BMI, body mass index; FSH, follicle-stimulating hormone; LH, luteinizing hormone; To, testosterone; others including mild ovarian stimulation protocol, luteal-phase ovarian stimulation protocol, natural protocol etc. *Both fresh embryo transfer cycle and frozen embryo transfer cycle were abandoned. Data are presented as mean±SD or median (interquartile range) for continuous variable and n(%) for categorical variable. Bold values represent statistical significance (p<0.05).

a Statistically significant differences between superabundant oocyte factor and relative male factor.

b Statistically significant differences between superabundant oocyte factor and absolute male factor.

c Statistically significant differences between relative male factor and absolute male factor.

**Table 2** shows the laboratory and pregnancy outcomes of oocyte warming cycles in patients with different indications for oocyte cryopreservation. The oocyte survival rate of SOC was superior to absolute-MOC (88.95% vs 84.99%, P<0.001); however there was no difference between SOC and relative-MOC (88.95% vs 84.17%, P=0.329). The D3 high-quality embryo rate was higher in absolute-MOC than SOC (31.22% vs 23.68%, P<0.001). No significant differences were found in the clinical pregnancy rate, early miscarriage rate, or live birth rate among the three groups for fresh and frozen embryo transfer after oocyte warming. The CLBR was highest in absolute-MOC, and lowest in SOC (39.0% vs 28.9%, P=0.006).

**Table 2:** Pregnancy outcomes of oocyte warming cycles in patients with different indications for oocyte cryopreservation.

	SOC (*n* = 429)	Relative MOC (*n* = 90)	Absolute MOC(*n* = 272)	*p* value^①^	*p* value^②^	*p* value^③^
Oocytes warmed ^bc^	7.38±3.43	7.21±4.36	9.29±4.49	0.727	**<0.001**	**<0.001**
Survival oocytes, (%)^bc^	6.54±3.23(88.95)	6.10±4.00(84.17)	7.90±4.32(84.99)	0.329	**<0.001**	**0.001**
Fertilization rate (%)	65.80(63.24-68.36)	67.47 (60.93-74.01)	66.19(63.07-69.30)	0.604	0.851	0.701
D3 high-quality embryo rate ^b^	23.68(20.81-26.55)	29.09(22.31-35.87)	31.22(27.77-34.67)	0.128	**<0.001**	0.580
Transfer embryos ^bc^	1(0, 2)	1(0, 2)	2(0, 2)	0.923	**0.003**	**0.044**
Frozen embryos ^bc^	0(0, 0)	0(0, 1)	0(0, 1)	0.088	**<0.001**	**0.038**
Perm sources ^abc^				**<0.001**	**<0.001**	**<0.001**
Husband semen	346(80.7)	85(94.4)	137(50.4)			
Husband PESA/TESA sperm	18(4.2)	5(5.6)	59(21.7)			
Donor frozen sperm	65(15.2)	0	76(27.9)			
Fresh embryo transfer cycle	N=245	n=53	n=186			
Clinical pregnancy rate, n(%)	105(42.9)	22(41.5)	89(47.8)	0.857	0.302	0.414
Early miscarriage rate, n(%)	10(9.5)	5(22.7)	13(14.6)	0.106	0.275	0.371
Live birth rate, n(%)	91(37.1)	17(32.1)	73(39.2)	0.487	0.656	0.342
Frozen embryo transfer cycle	n=69	n=21	n=73			
Clinical pregnancy rate, n(%)	38(55.1)	12(57.1)	41(56.2)	0.867	0.896	0.936
Early miscarriage rate, n(%)	3(7.9)	1 (8.3)	6(14.6)	0.961	0.341	0.553
Live birth rate, n(%)	34(49.3)	11(52.4)	34(46.6)	0.803	0.748	0.639
Cumulative live birth rate, n(%) ^b^	124(28.9)	27(30.0)	106(39.0)	0.835	**0.006**	0.126

Data are presented as mean±SD or median (interquartile range) for continuous variable and n(%) for categorical variable. Bold values represent statistical significance (*p*<0.05).

^a^ Statistically significant differences between superabundant oocyte factor and relative male factor

^b^ Statistically significant differences between superabundant oocyte factor and absolute male factor

^c^ Statistically significant differences between relative male factor and absolute male factor.

**Figure 1:**
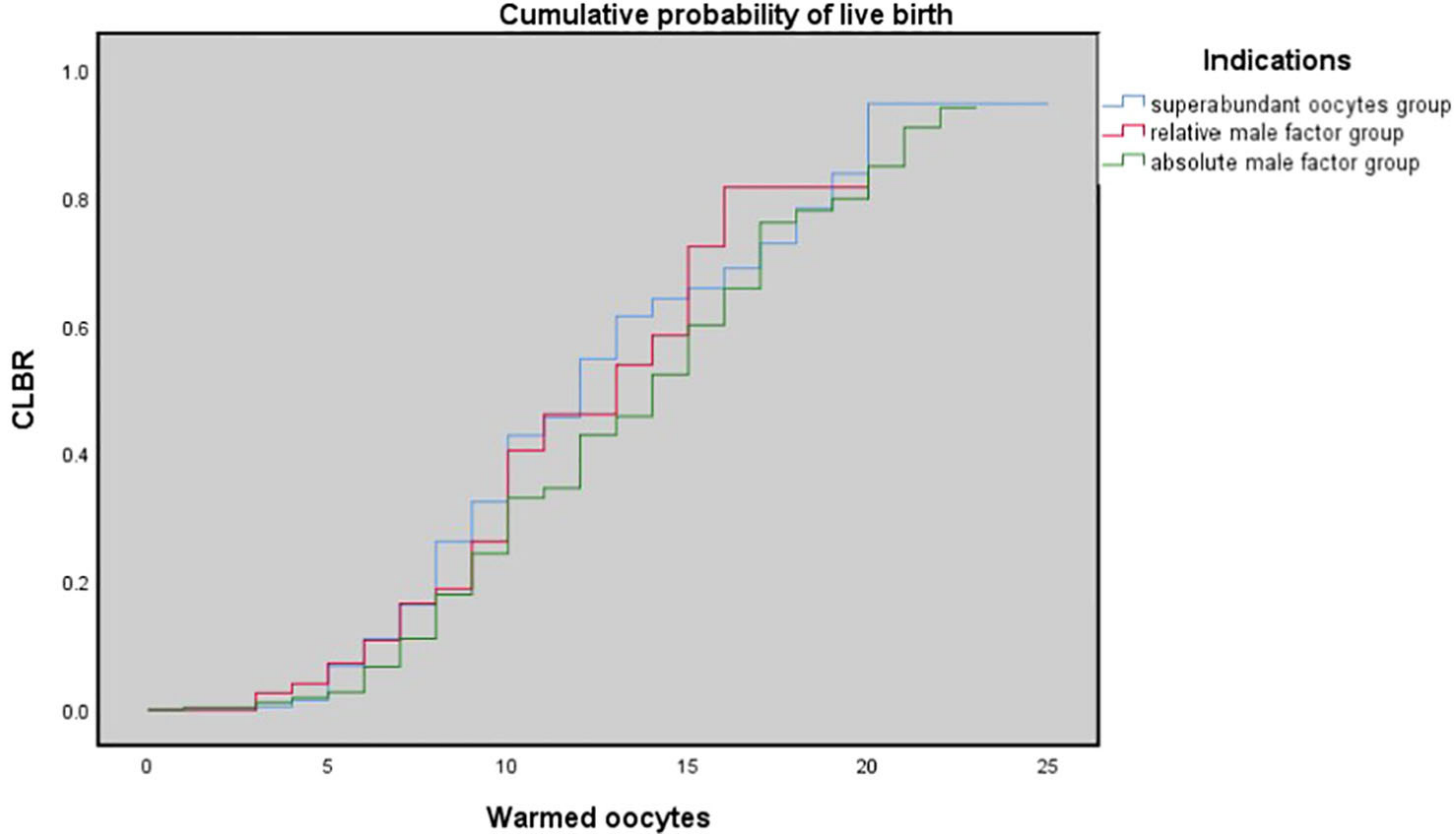
The cumulative probability of live birth according to number of oocytes thawed. Kaplan-Meier plotting of cumulative probability of live birth (CLBR) for the superabundant oocyte group, relative male factor group, absolute male factor group according to the number of oocytes warmed. Log-rank (Mantel-Cox); P = 0.092; Breslow (generalized-Wilcoxon); P = 0.051; and Tarone-Ware; P = 0.049.

**Figure 2:**
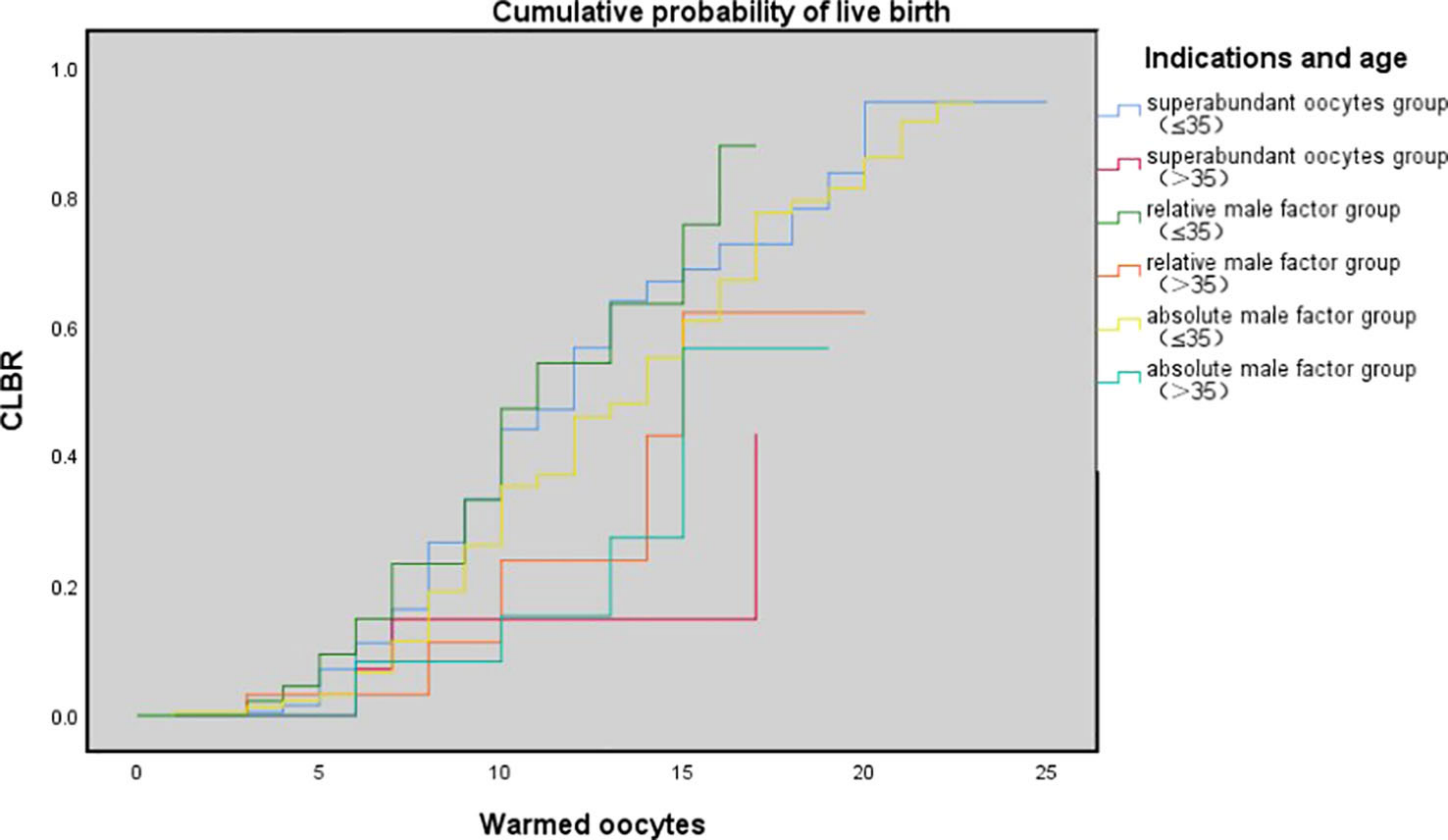
The cumulative probability of live birth according to age (≤35 vs. >35 y) and number of oocytes thawed. Kaplan-Meier plotting of cumulative probability of live birth (CLBR) for superabundant oocyte group, relative male factor group, absolute male factor group according to the number of oocytes warmed and patients' age at vitrification (≤ 35 year and >35 y). Log-rank (Mantel-Cox); P= 0.008; Breslow (generalized-Wilcoxon); P=0.023; and Tarone-Ware; P = 0.010.

**Table 3** shows the results of multivariate logistic regression analysis; absolute-MOC was used as a reference. After adjustment for age, BMI, preservation duration, use of donor sperm or not, use of PESA/TESA or not, number of oocytes retrieved, number of oocytes thawed, and oocyte survival rate, we found no correlation between indications for oocyte vitrification and CLBR. The Kaplan-Meier analysis showed no significant difference in CLBR between the three groups as the number of oocytes consumed increased (Log-rank (Mantel-Cox); P=0.092; Breslow (generalized-Wilcoxon); P=0.051; and Tarone–Ware; P=0.049) (**Figure 1**). The CLBR improved when more oocytes were warmed. **Figure 2** shows that the CLBR improved when more oocytes were consumed, and that older patients (≥35 y) had a lower CLBR than younger patients.

**Table 3:** Multivariate logistic regression for cumulative live birth rate.

	Crude OR (95%CI)	P-value	Adjusted OR (95%CI)	P-value
Age	0.914(0.883-0.946)	**<0.001**	0.920 (0.885-0.957)	**<0.001**
BMI	0.942(0.904-0.983)	0.006	0.954 (0.912-0.998)	**0.041**
Indications for oocyte vitrification				
absolute male oocyte factor*	1	——	1	——
superabundant oocyte factor	0.637 (0.462-0.878)	**0.006**	0.708(0.426-1.176)	0.182
relative male factor	0.671 (0.402-1.120)	0.127	1.700(0.924-3.128)	0.088
Preservation duration	1.011 (1.001-1.021)	**0.038**	1.009(0.998-1.021)	0.109
Donor frozen sperm				
No*	1	——	1	——
Yes	2.040 (1.407-2.958)	**<0.001**	2.249(1.445-3.502)	**<0.001**
Husband PESA/TESA sperm				
No*	1	——	1	——
Yes	1.022 (0.628-1.663)	0.929	0.878(0.502-1.535)	0.648
Oocytes retrieved	1.029(1.012-1.047)	**0.001**	1.007(0.974-1.041)	0.688
Warmed oocytes	1.144(1.101-1.189)	**<0.001**	1.126(1.063-1.192)	**<0.001**
Frozen oocyte survival rate	3.366 (1.431-7.917)	**0.005**	5.979(2.263-15.801)	**<0.001**

Bold values represent statistical significance (p<0.05).

**Table 4** shows the pregnancy outcomes of oocyte thawing cycles in patients with different indications for oocyte freezing. There were no differences in the rates of gestational diabetes mellitus, hypertension during pregnancy, caesarean section, new-born gender, birth weight, and gestational age after fresh ET and frozen embryo transfer (FET) between the three groups. The neonatal defect rates for singleton live births after ET in the three groups were 4.6%, 7.7%, and 5.7%, respectively; there were no differences among the three groups. There was no difference in neonatal defect rates between the three groups for twin live births after ET (5.8% vs 12.5%, 5.8% vs 2.5%, P=0.343). In relative-MOC, all singleton live births after FET were healthy. The rate of neonatal defects was the same in SOC and absolute-MOC, both at 5.9%. The pregnancy outcomes of ET and FET after oocyte warming are shown in **Supplementary Table 1**. The rate of twin pregnancies was higher in the ET group than in the FET group. The rates of gestational diabetes mellitus, hypertension during pregnancy, caesarean section, new-born gender, birth weight, and gestational age were comparable between the two groups.

**Table 4:** Pregnancy outcomes of oocyte thawing cycles in patients with different indications for oocyte freezing.

	SOC (*n* = 429)	Relative MOC (*n* = 90)	Absolute MOC(*n* = 272)	*p* value
Fresh embryo transfer cycle				0.912
Singleton livebirth cycles, n(%)	65(71.4)	13(76.5)	53(72.6)	
Twin livebirth cycles, n(%)	26(28.6)	4(23.5)	20(27.4)	
Number of babies born, n				0.107
Male, n(%)	50(42.7)	14(66.7)	47(50.5)	
Female, n(%)	67(57.3)	7(33.3)	46(49.5)	
Singleton livebirth				
Gestational diabetes mellitus, n(%)	3(4.6)	2(15.4)	1(1.9)	0.113
Hypertension during pregnancy, n(%)	6(9.2)	0	6(11.3)	0.679
Mode of delivery, n(%)				0.660
Vaginal	15(23.1)	3(23.1)	16(30.2)	
Caesarean delivery	50(76.9)	10(76.9)	37(69.8)	
Gestational age (weeks)	39.27±1.17	39.65±1.08	39.20±2.07	0.642
Gestational age category, n(%)				0.557
<37 weeks	2(3.1)	0	3(5.7)	
37-41 weeks	59(90.8)	11(84.6)	44(83.0)	
≥41 weeks	4(6.2)	2(15.4)	6(11.3)	
Weight category, n(%)				0.855
SGA	2(3.1)	0	2(3.8)	
LGA	22(33.8)	5(38.5)	14(26.4)	
Congenital defect(%)	3(4.6)	1(7.7)	3(5.7)	0.858
Twin livebirth				
Gestational diabetes mellitus, n(%)	0	0	0	
Hypertension during pregnancy, n(%)	4(15.4)	1(25.0)	1(5.0)	0.235
Mode of delivery, n(%)				0.111
Vaginal	0	0	3(15.0)	
Caesarean delivery	26(100)	4(100)	17(85.0)	
Gestational age (weeks)	36.20±1.61	37.93±1.17	36.40±1.84	0.139
Gestational age category , n(%)				**0.048**
<37 weeks	17(65.4)	0	11(55.0)	
37-41 weeks	9(34.6)	4(100)	9(45.0)	
≥41 weeks	0	0	0	
Weight category , n(%)				0.583
SGA	8(15.4)	0	8(20.0)	
LGA	4(7.7)	0	1(2.5)	
Congenital defect(%)	3(5.8)	1(12.5)	1(2.5)	0.343
Frozen embryo transfer cycle				0.622
Singleton livebirth cycles, n(%)	34(100)	11(100)	34(94.4)	
Twin livebirth cycles, n(%)	0	0	2(5.6)	
Number of babies born, n				0.541
Male, n(%)	17(50.0)	4(36.4)	21(55.3)	
Female, n(%)	17(50.0)	7(63.6)	17(44.7)	
Singleton livebirth				
Gestational diabetes mellitus, n(%)	3(8.8)	0	0	0.201
Hypertension during pregnancy, n(%)	3(8.8)	0	2(5.9)	0.846
Mode of delivery, n(%)				0.257
Vaginal	6(17.6)	3(27.3)	12(35.3)	
Caesarean delivery	28(82.4)	8(72.7)	22(64.7)	
Gestational age (weeks)	39.14±1.22	39.48±1.14	39.40±1.32	0.576
Gestational age category, n(%)				0.588
<37 weeks	2(5.9)	1(9.1)	1(2.9)	
37-41 weeks	31(91.2)	9(81.8)	30(88.2)	
≥41 weeks	1(2.9)	1(9.1)	3(8.8)	
Weight category, n(%)				0.653
SGA	0	0	1(2.9)	
LGA	9(26.5)	4(36.4)	7(20.6)	
Congenital defect(%)	2(5.9)	0	2(5.9)	1.000

SGA, small for gestational age; LGA, large for gestational age. Data are presented as mean±SD or median (interquartile range) for continuous variable and n(%) for categorical variable. Bold values represent statistical significance (*p*<0.05).

## 4 Discussion

Oocyte cryopreservation techniques have changed from slow freezing to vitrification owing to safety and efficacy over the last decade (12), and oocyte vitrification has been gradually introduced into assisted reproduction treatment in a variety of clinical scenarios (13). The aim of this study was to evaluate the effectiveness and safety of supernumerary autologous oocyte vitrification. In the present study the CLBR of oocyte warming cycles for SOC was compared with cycles for MOC. Women who underwent oocyte cryopreservation owing to a surplus of retrieved oocytes were found to have similar CLBR to those for male factors, and the CLBR improved as the number of oocytes consumed increased. Neonatal outcomes did not show a significant difference among different indication groups.

In 2003, the European Society of Human Reproduction and Embryology stated that the purpose of reproductive medicine is to help couples with an unfulfilled wish to have a healthy child (14). Effectiveness refers to how well a treatment works in clinical practice and the extent to which it helps patients achieve their desired goal of having a healthy baby. So, to increase the effectiveness of fertility treatment, it is necessary to increase the chances of a couple having a baby. Therefore, for couples with a high number of retrieved oocytes, does vitrifying a small number of oocytes diminish the chance of having a baby? Although there is a lack of randomized controlled studies (RCT), our present data may confirm the answer to some extent. From 2013–2019, 4536 women had their supernumerary oocytes cryopreserved. During the same period, the clinic treated 429 women who had stored surplus oocytes, and a small part of those (42 women) had given birth to one live baby following previous fresh oocyte transfer. Therefore, it was estimated that around 90% of patients delivered a live baby from fresh oocytes. This confirmed the effectiveness of the clinical strategy of vitrifying a small number of oocytes when superabundant oocytes were retrieved.

However, for the remaining approximately 9% of patients, who failed to have a live birth with their fresh oocytes, the question exists: is worth spending more time, emotion, and money on the remaining cryopreserved oocytes? Through a rough comparison, we found that SOC patients had the lowest CLBR per cycle, while absolute-MOC had the highest CLBR (28.9% vs. 39%, P=0.006). However, there were significant differences in basic characteristics among the three groups, including age and the number of warmed oocytes. After adjustment for confounding factors, the indications for oocyte cryopreservation did not have an impact on CLBR. The key influencing factors were age, BMI, use of donor sperm, number of warmed oocytes, and oocyte survival rate. This outcome provided certain positive information, but more importantly, patients need to be informed of the probability of having a baby using their currently cryopreserved oocytes. In the present study, the oocyte-to-baby rates in SOC, relative-MOC, and absolute-MOC were 4.77%, 5.4%, and 5.2%, respectively. However, the individual variations were diverse, and an average percentage achieved by the simple division of the number of babies by the number of oocytes does not represent the real situation. Obviously, the trend of CLBR increased according to the number of oocytes consumed. However, as shown by the curve, this relationship is not linear, as a powerful confounding factor, age has an impact on oocyte quality and chromosomal euploidy. Therefore, using the Kaplan–Meier analysis to assess the CLBR of oocytes consumed according to age is an accurate evaluation method. The current findings indicated that young SOC patients (≤35 y) could achieve reasonably successful CLBRs of 26.7% and 43.9% with 8 and 10 oocytes, respectively. There was a huge difference in CLBR when only 5 oocytes (7.1%) and when 15 occytes (68.6%) were used in young SOC patients, which suggests an approximately 6% increase in CLBR per additional oocyte for women ≤35 y in SOC. Results indicated that patients ≤35 y with 8–10 supernumerary cryopreserved oocytes could obtain a reasonable success rate even after failures with around 15 fresh oocytes. The same trend was observed in relative-MOC and absolute-MOC, and there was no significant difference in K-M analysis among the three groups. There were shortcomings for patients in each group: SOC had inferior embryo quality, relative-MOC had advanced age, and obviously absolute-MOC had male factors. However, there was a significantly different probability of having a baby according to the number of oocytes consumed when the ≤35 y and >35 y groups were compared in K-M analysis. Patients aged >35 y need more oocytes to achieve comparable outcomes to those in young women, but they never reach the highest outcomes achievable by the young groups because a plateau is reached much earlier in older women.

Studies comparing outcomes between fresh and vitrified sibling oocytes have also been published. Most studies show decreased embryo quality in a vitrified group including our previous clinical research (15). Furthermore, vitrified oocytes can achieve higher live birth rates when a live birth has already been obtained from their cohort of fresh oocytes (16). These reports are in contrast to the current findings, and possible reasons were investigated that might explain why a few vitrified oocytes could achieve more live births than a large number of fresh sibling oocytes in populations of the present study. Firstly, during a vitrification-warming procedure oocytes will suffer hardening of the zona pellucida and need to be fertilized by ICSI; this strategy would be superior to cycles that failed with fresh oocytes as it overcomes fertilization disorders during IVF. The second advantage for vitrification-warmed oocytes is that embryo transfer into the endometrium of prepared cycles yields better implantation rates than in hyper-ovarian response stimulation cycles. Furthermore, warmed-oocyte cycles often give patients the opportunity to transfer fresh embryos at the cleavage stage, which offers the chance of implantation after repeated failure of vitrified blastocyst transfers. Thirdly, there is always a debate around whether obtaining a large number of oocytes affects oocyte quality or not. A recent study showed a negative correlation between oocyte number and maturation rate (17). Therefore, the additional incubation time after warming might have enhanced cytoplasmic maturation of vitrified oocytes from the SOC group. Finally, it is suggested that Ca_2_+ oscillations induced by the high concentration of cryoprotectants in the vitrification solution could be responsible for both the higher rates of *in vitro* maturation and the improved subsequent embryonic development in immature human oocytes (18). As a result, some poor quality oocytes may benefit from the Ca_2_+ oscillations produced by osmotic-shock during the vitrification-warming process, in a similar way to the technology of artificial activation that researchers have recently shown to increase embryo quality in poor quality oocytes (19, 20). These possible reasons might explain why in the special population of SOC group, the vitrified oocytes achieved more live births than the fresh oocytes.

Safety is “the state of being protected from danger or harm, or the condition of not being likely to cause damage or harm”. For oocyte vitrification, safety refers to the possible negative consequences of a treatment to the mother or her offspring, occurring either directly as a consequence of the treatment itself, as either complications of pregnancy or as an impact of the treatment on the long-term health of the mother or child. Previous studies have focused the safety of vitrification of oocytes on the oocyte freezing technology itself. Previous studies have found that dimethyl sulfoxide has no genotoxicity, while ethylene glycol has indirect genotoxicity due to the addition of exogenous cytochrome P-450 oxidation system, but propylene glycol always has genotoxicity (21). There are also studies on the damage of oocyte structures and function, like meiotic spindle and mitochondria, caused by freezing process. In 2014, Cobo and his colleagues compared 1,027 newborns born with vitrified oocytes with 1,224 newborns born with fresh oocytes. By excluding confounding factors, vitrified oocytes group had no adverse effects on pregnancy complications, neonatal gestational weeks, birth weight, Apgar score, birth defects and perinatal mortality (22). Another study analyzed reports on obstetric outcomes of frozen oocytes from 1986 to 2008, including 936 infants born with frozen oocytes (slow freezing and vitrification), and 1.3% of them had congenital malformations, which was similar to the congenital malformation rate of infants born with natural conception during the same period (23). Previous studies have provided considerable evidence for obstetric and neonatal outcomes of vitrified oocytes, but it has not been confirmed whether oocytes cryopreserved due to surplus oocytes are equally safe. Therefore, we compared the obstetric and neonatal outcomes the three groups. The preliminary data of safety in our article showed no significant differences in the rates of gestational diabetes mellitus, hypertension during pregnancy, caesarean section, new-born gender, birth weight, and gestational age after ET and FET between the SOC and MOC groups. The primary concern of clinicians and patients is the health of new-borns (22, 24), even though oocytes are known to be susceptible to cryodamage to some extent (24). In the current study, no differences were found in the incidence of congenital malformations between the SOC and MOC groups. For further study, the pregnancy outcomes of ET and FET from vitrified oocytes were compared. The rate of twin pregnancies was found to be higher in the ET group than in FET; however, the rates of gestational diabetes mellitus, hypertension during pregnancy, caesarean section, new-born gender, birth weight, gestational age, and congenital defects were comparable between the two groups. This could further indicate that double-freezing procedures do not increase the risk of obstetric and neonatal outcomes. Our preliminary data of safety shows that after the failure of a number of fresh oocytes to produce a live born, sibling supernumerary oocytes that had been vitrified were found to be equally as safe as vitrified oocytes from other indications. In the future, a large-scale study with long-term follow-up is needed to estimate the children growth outcomes for more information of safety of oocyte vitrification application in ART cycles.

There are few and inconsistent reports about the effectiveness and safety of autologous oocyte vitrification in China. Previous studies have focused on the effectiveness and safety of the oocyte vitrification technique itself (25). The focus of the present study was the evaluation of a special patient population who mostly had failed to achieve a live birth from fresh oocytes and came back to use sibling supernumerary vitrified oocytes. A comparison with other oocyte vitrification indications could be informative for clinicians, although, most oocyte vitrification procedures in clinical circumstances could not be evaluated by prospective randomized controlled trials for ethical and legal reasons. A further benefit of the present study was the relatively stable laboratory team with experienced technicians in oocyte vitrification. As a retrospective study, there was inevitably selection bias in this study, and all confounders could not be introduced into the multivariable regression analysis. And for the peculiar population (vitrified oocytes due to various unexpected reasons in the routine IVF/ICSI cycles) involved in present study, the sample size is small. Secondly, the CLBR was influenced not only by oocyte quality but also by the quality of the male partner’s sperm. Given the different indications for oocyte cryopreservation, the male partner factors in this study varied widely and did impact the results. For this reason, sperm source was included as a potential confounder in the regression model to reduce the interference.

## 5 Conclusions and perspectives

When few live births are obtained from fresh oocytes, a relatively ideal CLBR can still be achieved from any remaining sibling vitrified oocytes. The CLBR of vitrified oocytes for different indications was correlated with age and number of warmed oocytes. For women who have plenty oocytes retrieved, the strategy of cryopreserving a small number of oocytes is a valuable option and might benefit them in the future. Additional data from autologous oocyte vitrification research employing a large-scale and variable-controlled methodology with extending follow-up will complement and clarify the current results.

## Author contributions

SG contributed to design of the study and editing. XF wrote the main manuscript text and analysed the data. YZ, ShulG, ShuzG and MZ contributed to the review and the version of the manuscript. JM and Z-JC conducted a general review of manuscript. All authors contributed to the article and approved the submitted version.
