# Supplementary figures and images for: Efficiency and safety of vitrification of surplus oocytes following superovulation: a comparison of different clinical indications of oocyte cryopreservation in IVF/ICSI cycles
Source: Front Endocrinol (Lausanne). 2023 Oct 5;14:1221308. doi: 10.3389/fendo.2023.1221308 (PMC10585140; doi:10.3389/fendo.2023.1221308)

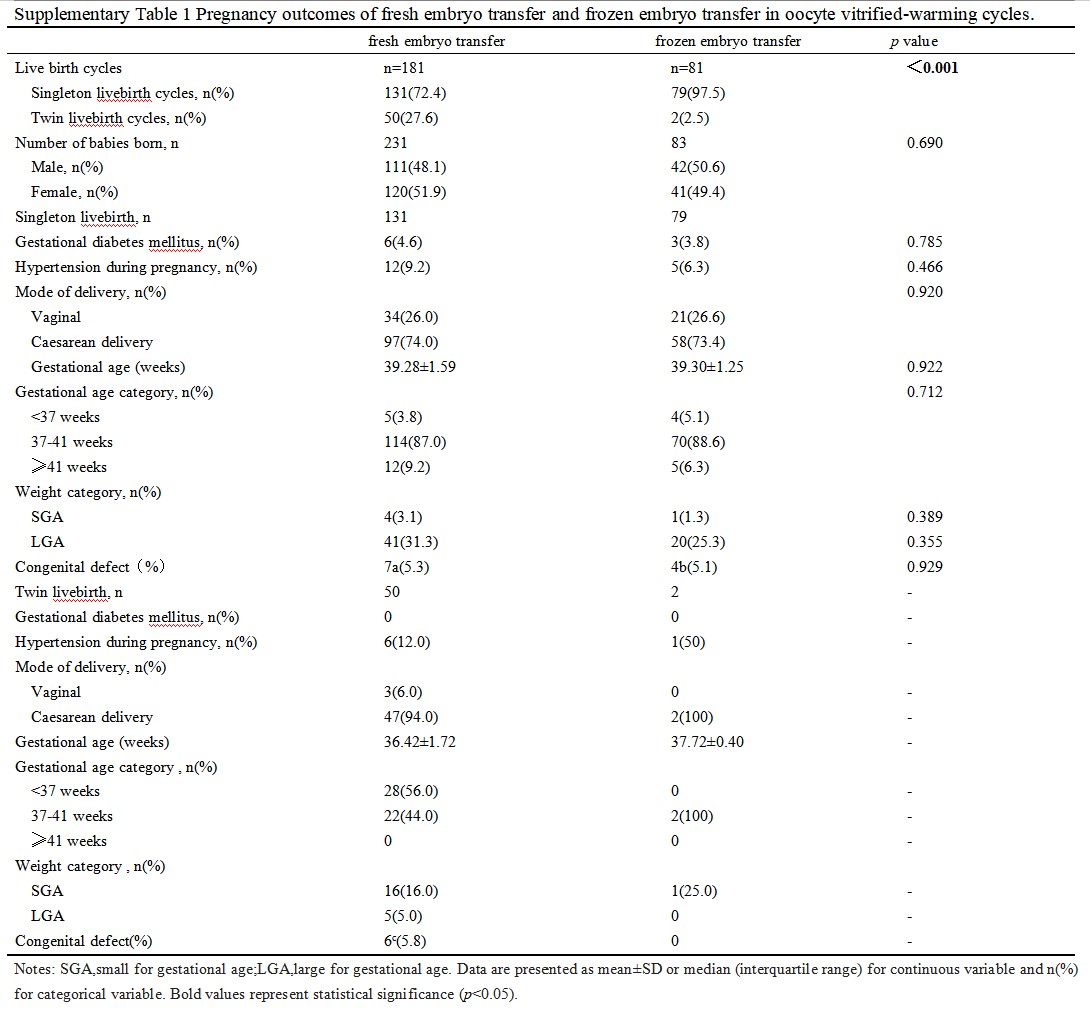

Supplement: Supplementary file 1 [file Image_1.jpeg]
